# A Synbiotic Formulation Comprising *Bacillus subtilis* DSM 32315 and L-Alanyl-L-Glutamine Improves Intestinal Butyrate Levels and Lipid Metabolism in Healthy Humans

**DOI:** 10.3390/nu14010143

**Published:** 2021-12-29

**Authors:** Heike tom Dieck, Christiane Schön, Tanja Wagner, Helga Carola Pankoke, Monika Fluegel, Bodo Speckmann

**Affiliations:** 1 Evonik Operations GmbH, Rodenbacher Chaussee 4, D-63457 Hanau, Germany; heike.tom-dieck@evonik.com (H.t.D.); helga-carola.pankoke@evonik.com (H.C.P.); monika.fluegel@evonik.com (M.F.); bodo.speckmann@evonik.com (B.S.); 2 BioTeSys GmbH, Schelztorstrasse 54-56, D-73728 Esslingen, Germany; t.wagner@biotesys.de

**Keywords:** probiotic, colon-targeted delivery, dietary supplement, metabolic syndrome, microbiome modulation

## Abstract

The gut microbiota is a crucial modulator of health effects elicited by food components, with SCFA (short chain fatty acids), especially butyrate, acting as important mediators thereof. We therefore developed a nutritional synbiotic composition targeted at shifting microbiome composition and activity towards butyrate production. An intestinal screening model was applied to identify probiotic *Bacillus* strains plus various amino acids and peptides with suitable effects on microbial butyrate producers and levels. A pilot study was performed to test if the synbiotic formulation could improve fecal butyrate levels in healthy humans. A combination of *Bacillus subtilis* DSM (Number of German Collection of Microorganisms and Cell Cultures) 32315 plus L-alanyl-L-glutamine resulted in distinctly increased levels of butyrate and butyrate-producing taxa (*Clostridium* group XIVa, e.g., *Faecalibacterium prausnitzii*), both in vitro and in humans. Moreover, circulating lipid parameters (LDL-, and total cholesterol and LDL/HDL cholesterol ratio) were significantly decreased and further metabolic effects such as glucose-modulation were observed. Fasting levels of PYY (Peptide YY) and GLP-1 (Glucagon-like Peptide 1) were significantly reduced. In conclusion, our study indicates that this synbiotic composition may provide an effective and safe tool for stimulation of intestinal butyrate production with effects on e.g., lipid and glucose homeostasis. Further investigations in larger cohorts are warranted to confirm and expand these findings.

## 1. Introduction

The gastrointestinal microbiota forms an intriguing and interconnected relationship with orally ingested matter—be it food or pharmaceutical ingredients—and human physiology. In that sense, microbiota composition and activity are affected by diet, and on the other hand dietary molecules are converted through a plethora of (microbe-specific) metabolic pathways to a partly absorbable metabolome. Examples of gut microbiota-derived metabolites with known effects on the host include phenolic acids, indole derivatives, and short-chain fatty acids (SCFA) acetate, propionate, and butyrate. Butyrate is an important energy source and differentiation factor for colonic epithelial cells, it also supports the formation of mucin as well as tight junction proteins and thereby contributes to intestinal barrier integrity [1]. Furthermore, butyrate can trigger anti-inflammatory signaling via binding to arylhydrocarbon, G protein-coupled receptor (GPR) 41, GPR109, and Peroxisome proliferator-activated receptor γ (PPARγ) receptors and has consequently been related to immune and metabolic functions in the gut and beyond, including the brain. A fraction of around two percent of luminal butyrate enters the circulation [2] via the portal vein, and it thereby also affects tissues beyond the gastrointestinal tract, such as the liver, adipose tissue, and pancreas. The systemic actions of a sufficient butyrate supply can be summarized as a (beneficial) modulation of glucose and lipid metabolism leading to increased insulin sensitivity, reduced plasma glucose and cholesterol levels, as well as increased satiety and decreased blood pressure [3]. Although available studies have not established a causal role for butyrate or other SCFA in the pathophysiology of metabolic disorders, it appears likely that they at least partly contribute to the health effects caused by e.g., fiber-rich diets [4,5]. In conclusion, an increase of colonic butyrate levels is an attractive target for the development of microbiota-targeted intervention strategies to achieve targeted health outcomes, particularly for the prevention and adjuvant treatment of inflammatory bowel diseases (IBD) and metabolic disorders such as type 2 diabetes, traits of the metabolic syndrome, and cardiovascular diseases.

Colonic butyrate levels can be targeted by means of prebiotics, probiotics, combinations thereof and by direct intake of butyric acid in the form of salts or butyrate precursors, such as tributyrin. Sodium butyrate has been clinically assessed as a co-treatment for IBD patients, e.g., supporting the efficacy of 5-aminosalicylic acid (5-ASA) in refractory distal ulcerative colitis in a topical application [6]. However, convenient and patient-friendly application forms are crucial compliance factors, even more so in a preventative approach, hence oral interventions are favorable. For butyrate this has been realized by a delayed-release coated tablet formulation targeting butyrate to the distant small intestine/colon [7]. The kinetic profile indicated a sudden intraluminal release of butyrate from the carrier, which supposedly is disadvantageous over a more sustained uptake of butyrate formed continuously within the gastrointestinal tract. Moreover, the intense odor of butyrate-containing supplements limits their applicability.

Probably the most well-established means of triggering butyrate formation is the ingestion of prebiotic fibers, either through the normal diet or via fortified foods or dietary supplements. The most investigated and applied prebiotics are fructo-oligosaccharides (FOS), galacto-oligosaccharides (GOS), arabinoxylan-oligosaccharides (AXOS), xylo-oligosaccharides (XOS), and beta-glucans. These carbohydrates form part of the larger group of FODMAPs (fermentable oligosaccharides, disaccharides, monosaccharides, and polyols). Diets low in FODMAP content have become increasingly popular [8], as FODMAPs often cause unwanted side-effects, e.g., diarrhea, constipation, and flatulence. A low FODMAP diet effectively reduces these symptoms in patients with irritable bowel syndrome [9]. The use of fibers as source of intestinal SCFA is therefore of limited applicability at least for certain populations.

An alternative approach is the direct application of butyrate-producing bacteria as supplemental or therapeutic probiotics. The human gut microbiota is known to use four catabolic pathways that lead to butyrate [10], with three of them processing proteins/amino acids as educts, and the pyruvate/acetyl-CoA pathway (Ac pathway) processing proteins as well as carbohydrates, the latter being the most abundant in a multi-ethnic metagenome analysis [11]. *Faecalibacterium prausnitzii*, *Oscillibacter*, and *Clostridium* XIVa, comprise the dominant core community of all bacteria associated with the Ac pathway. The species of these taxa do not belong to the commonly used probiotic bacteria of the genera *Lactobacillus*, *Bifidobacterium*, and *Bacillus*, but have more recently been studied and applied as so-called next-generation probiotics, e.g., *Akkermansia muciniphila*, *Faecalibacterium prausnitzii*, *Clostridium butyricum*, *Clostridium beijerinckii*, and *Eubacterium hallii*. Though these developments are clearly interesting, a recent pilot trial assessing a cocktail including four of the above-mentioned next-generation probiotic strains failed to achieve a significant induction of fecal butyrate levels [12]. Another *Clostridium butyricum* strain has been characterized as a butyrate-producer in vitro and in rodents, but its butyrate-producing capacity in the human gut remains to be established [13]. Likewise, a 4-week intervention with 10^8^ CFU/day of a *Butyricicoccus pullicaecorum* strain had no effect on fecal butyrate levels [14]. Overall, the application of next generation/butyrate producing probiotics in humans is intriguing but also facing technical challenges, due to their strict anaerobic nature, making them difficult to produce and ensure long-term stability in formulated finished products.

Conclusively, there is a high interest for novel microbiota-targeted strategies to boost butyrate production and leverage subsequent health effects. As probiotic-only approaches have failed to increase fecal butyrate levels, we here followed a synbiotic strategy to achieve this target. Based on the fact that several *Bacillus* species have been reported to trigger gut microbial butyrate production indirectly [15,16], we aimed to identify a robust strain with documented probiotic functionalities [17,18] that can modulate the human colonic microbiota towards a pro-butyrogenic shift in its composition and activity, and to combine it with a stable, non-fiber substrate that bacteria can selectively use for butyrate production. The use of L-alanyl-L-glutamine (Ala-Gln) and related peptides and amino acids was based on their known catabolic routes towards butyrate, in the case of clostridium group XIV for the acetyl CoA butyrate pathway (Ac pathway). We here applied intestinal screening models for selection of most suitable pro- and prebiotics, followed by co-cultivation experiments and application of the most successful combination together with vitamin B12 as essential co-factor of the Ac pathway in a capsule formulation in a human pilot study, confirming its butyrate-producing efficacy in vivo.

## 2. Materials and Methods

### 2.1. In Vitro Colonic Screening Platform

TNO’s in vitro colonic screening platform (I-screen) was applied to screen a selection of amino acids, peptides, and probiotic strains for their potential to modulate gut microbiota composition and activity in vitro. The inoculum for the I-screen platform was prepared from fecal samples collected from six healthy adult volunteers (Caucasian with European lifestyle and nutrition, and no antibiotic usage in the last three months) as described elsewhere [19]. The pooling approach limited inter-individual variations and increased the probability to have a larger representation of bacterial species present in the human colon.

#### 2.1.1. Testing of Compounds in I-Screen

For the I-screen fermentations, the precultured standardized fecal inoculum was diluted 50 times in SIEM according to [16] with following modifications: pectin (0.047 g/L), xylan (0.047 g/L), arabinogalactan (0.047 g/L), amylopectin (0.047 g/L), starch (0.392 g/L), casein (24.0 g/L), Bacto pepton (24.0 g/L), ox-bile (0.4 g/L), and cysteine (0.2 g/L). The pH of the medium was adjusted to 5.8.

Probiotics: *Bacillus subtilis* DSM 32315, and three other *Bacillus* spp. were precultured separately in 50 mL LB Kelly medium for 16 h. Incubation was done in shaking flasks at 37 °C under aerobic conditions. After incubation, bacterial density was determined by optical density measurement at 600 nm. A final stock solution of 1 × 10^10^ cells/mL was prepared in 1 mL buffer solution (0.1 mM MES pH 6). The suspension of each strain was introduced into the I-screen to a final level of about 10^9^ cells/mL and incubation was performed under microaerophilic conditions (0.2% O_2_, 0.2% CO_2_, 10% H_2_, 89.6% N_2_) at 37 °C at 300 rpm for 20 h. All experiments were carried out in triplicates.

Amino acids and dipeptides: The fecal inoculum was prepared as described above. Incubations of L-tryptophan, D-tryptophan, N-acetyl-trp, proline, ornithine-aspartate, Met-Met, Gly-Tyr, Gly-Gln, and Ala-Gln were performed under standard anaerobic conditions (0% O_2_, 10% CO_2_, 10% H_2_, 80% N_2_) at 37 °C at 300 rpm for 20 h. Except for proline (0.2 mg/mL), all substances were applied at a final concentration of 1.5 mg/mL. All experiments were carried out in triplicates.

#### 2.1.2. DNA Isolation

DNA extraction for the sequencing of 16S rRNA coding genes was performed as described by Ladirat et al. [19] with minor modifications. Approximately 100 μL of the culture materials were added to the wells of a 96-well plate containing per well 300 µL of lysis buffer (Mag Mini DNA Isolation Kit, LGC Ltd., Hoddesdon, UK), 500 μL zirconium beads (0.1 mm; BioSpec products, Bartlesville, OK, USA) and 500 μL of phenol saturated with Tris-HCl (pH 8.0; Carl Roth GMBH, Karlsruhe, Germany). The 96-well plate was placed in a Mini-BeadBeater-96 (BioSpec products, Bartlesville, OK, USA) for 2 min at 2100 oscillations/min. DNA was subsequently purified using the Agowa Mag Mini DNA Isolation Kit according to the manufacturer recommendations. Extracted DNA was eluted in a final volume of 60 μL buffer.

#### 2.1.3. V4 16S rRNA Gene Sequencing

The microbiota composition was analyzed by 16S rRNA gene amplicon sequencing of the V4 hypervariable region. This was achieved through a series of steps:

To determine the amount of bacterial DNA in the I-screen DNA samples, quantitative polymerase chain reaction (qPCR) was performed using primers specific for the bacterial 16S rRNA gene (Forward primer: CGAAAGCGTGGGGAGCAAA; Reverse primer: GTTCGTACTCCCCAGGCGG; Probe: 6FAM-ATTAGATACCCTGGTAGTCCA-MGB). Subsequently, using 500 pg of DNA [18], PCR amplicons of the V4 hypervariable region of the 16S rRNA gene were generated for each individual sample with F515/R806 primers [19]. Primers included Illumina adapters and a unique 8-nt sample index sequence key [18]. A mock control was included for technical quality control. The amount of amplified DNA per sample was quantified using the dsDNA 910 Reagent Kit on the Fragment Analyzer (Advanced Analytical). The amplicon libraries were pooled equimolarly and purified from 1.2% agarose gel using the Gel Extraction Kit (Qiagen, Venlo, Netherlands). The library was quantified using the Quant-iT™ PicoGreen^®^ dsDNA Assay Kit (Thermo Fisher Scientific, Landsmeer, Netherlands). Paired-end sequencing of amplicons was conducted on the Illumina MiSeq platform (Illumina, Eindhoven, The Netherlands).

The sequence data was processed with Mothur v.1.36.1 in line with the Mothur MiSeq SOP. Before merging the read pairs, low quality regions were trimmed using ‘Btrim’ (sliding window size = 5 nt, average quality score = 25). After merging, the sequences were filtered by length without allowing ambiguous bases. The unique sequences were aligned to the bacterial SILVA SEED reference alignment release 102 (available at: http://www.mothur.org/wiki/Silva_reference_files, accessed on 31 August 2021) and sequences that were too short were removed using ‘screen.seqs’ (parameters: “optimize = start-end, criteria = 90”). In each sample, chimeric sequences were identified using UCHIME [20] in de novo mode and removed. Next, sequences that occurred with less than 10 read counts in the whole dataset were removed. Taxonomy was assigned to all sequences using the Ribosomal Database Project (RDP) naïve Bayesian classifier with confidence threshold of 60% and 1000 iterations [21] and the Mothur-formatted version of the RDP training set v.9 (trainset9_032012). Sequences were grouped using Minimum Entropy Decomposition (MED) algorithm that clusters 16S rRNA gene amplicons in a sensitive manner [20]. To filter noise, the “minimum substantive abundance” filter was set to 200.

#### 2.1.4. Short Chain Fatty Acid and Lactate Analysis

I-screen supernatant samples were centrifuged (~4000× *g*, 5 min) and sterile-filtered (0.45 µm). A mixture of formic acid (20%), methanol and 2-ethyl butyric acid (internal standard, 2 mg/mL in methanol) was added. A 3 µL sample with a split ratio of 75.0 was injected on a GC-column (ZB-5HT inferno, ID 0.52 mm, film thickness 0.10 µm; Zebron; Phenomenex, Torrance, CA, USA) in a Shimadzu GC-2014 gas chromatograph. SCFA parameters analyzed were: acetic acid, propionic acid, iso-butyric acid, butyric acid, and iso-valeric acid. For the analysis of lactate content in the I-screen samples, samples were centrifuged (~4000× *g*, 5 min) and the clear supernatant was sterilized by using a 0.45 µm cellulose acetate membrane filter Z746487 (Whatman^®^, Cytiva, Little Chalfont, UK). The D- and L-lactate was determined by the Arena 20XL analyzer. The assay is based on the enzyme-catalyzed reaction of lactic acid + NAD ↔ Pyruvate + NADH. The amount of NADH generated is stoichiometrically linked to the amount of lactic acid present and is measured by the amount of light absorbed by NADH at a wavelength of 340 nm. By using both the L-lactate dehydrogenase and D-lactate dehydrogenase enzymes in separate reactions, the individual concentrations of L-lactic acid and D-lactic acid were determined.

#### 2.1.5. Data Analysis

Statistical analysis of the 16S RNA gene amplicon sequencing data was performed by using R version 3.3.2 (CRAN.R-project.org). The overview of all sample groups was created using non-metric multidimensional scaling as implemented in R package ‘*vegan*’. The multidimensional scaling procedure was applied to a Bray-Curtis distance matrix created using the *dist()* function of the ‘*proxy*’ package. Statistics on SCFAs and tryptophan metabolites were performed using linear mixed models as implemented in R ‘*base*’ Multivariate statistics on the 16S RNA gene amplicon sequencing data was performed using perMANOVA as implemented in the ‘*vegan*’ package. For the analysis of SCFA and changes of microbiome composition, the type of substance was used as a random factor in the analysis. For analysis of the effects of the *Bacillus* strains, all species of the genus *Bacillus* were removed from the 16S sequencing data and the data were rescaled and transformed using Wisconsin double transformation and square root transformation.

### 2.2. Human Study

The study was conducted as open-label, single center pilot study between October and December 2020 at an independent Nutritional CRO, BioTeSys GmbH, in Esslingen (Germany) after review by the Institutional Review Board of Landesärztekammer Baden-Württemberg (approval number: F-2020-042) and registration at the German Clinical Trials Register (DRKS, Freiburg, Germany; DRKS-ID: DRKS00023166). All subjects provided their written consent before participation and the principles of Good Clinical Practice and the Declaration of Helsinki were followed.

The objective of the study was to investigate the proof of concept of the study product on fecal SCFA levels over a four-week intake phase. Furthermore, the impact on gut microbiota composition, fasting glucose, lipids, and satiety hormones (total GLP-1, PYY) was investigated.

#### 2.2.1. Study Subjects

**Figure 1:**
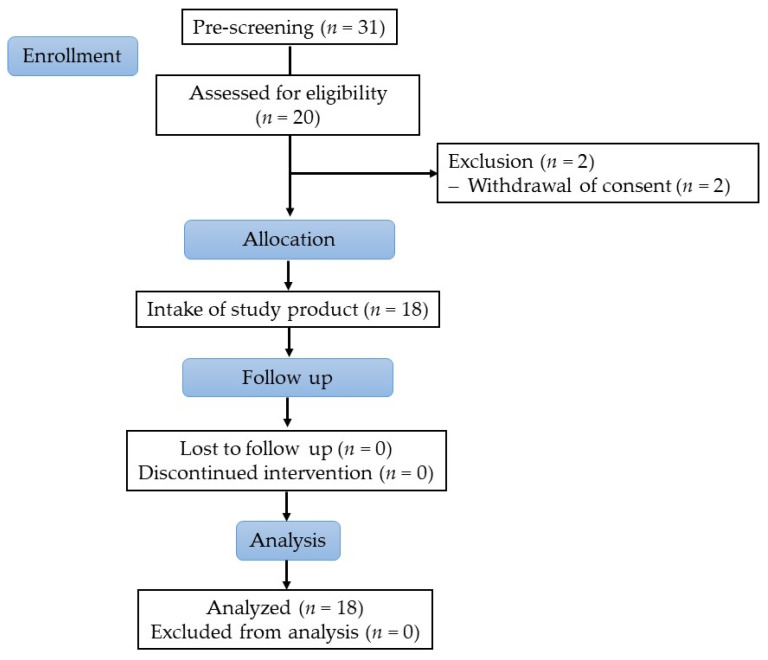
Study flow diagram.

Overall, 31 people were pre-screened for eligibility, from which 20 subjects were screened to ascertain their eligibility (Main inclusion criteria: healthy men between 18 and 40 years, body mass index (BMI) ≥ 19 and ≤ 30 kg/m², non-smoker. Main exclusion criteria: history or presence of any severe medical disorder potentially interfering with the study (e.g., mal absorption, chronic gastro-intestinal diseases, heavy depression, diabetes, acute cancers within last 3 years), subject under prescription for medication or taking dietary supplements possibly interfering with this study (such as anti-spasmodic, laxatives and anti-diarrheic drugs or other digestive auxiliaries, use of PPIs, bismuth salts and/or H_2_-antagonists, fibers etc.) within 2 weeks prior to study start or during the study, intake of antibiotics in the last 2 months, significant changes in lifestyle or medication (within last 2 months), vegetarians, vegans, subjects consuming food or drinks claimed as ‘probiotic’ or ‘prebiotic’ more than once weekly, consumption of more than three portions of fruits and vegetables (sum) per day, subjects with stool frequency of ≤2 stools per week). Finally, 18 subjects were enrolled, and all finished the study according to protocol, as shown in Figure 1.

#### 2.2.2. Study Design

Following a 2-week run-in phase (basal characterization of subjects with regard to bowel function), subjects supplemented the study product during an intervention period of 4 weeks. At baseline (visit 1), after 2 weeks (visit 2) and at the end of intervention (after 4 weeks, visit 3), subjects came to the study site and stool and blood samples were collected for biomarker determination and microbiome analysis.

#### 2.2.3. Study Product

The study product consisted of HPMC capsules comprising 2 × 10^9^ CFU *Bacillus subtilis* DSM 32315, 290 mg L-Alanyl-L-Glutamine, 90 mg Curcuma extract (approx. 70–80% Curcumin), 90 mg Green tea extract (approx. 50% EGCG), 5 mg Zinc, 0.56 mg Vitamin B6, 20 µg D-Biotin, 0.75 µg Vitamin B12, 4 µg Vitamin D, 2.4 mg Pantothenic acid (content per 2 capsules, size 1) (SAMANA^®^ Force, Evonik, Darmstadt, Germany). Vitamin B12 serves as cofactor of the enzyme glutamate mutase, which catalyzes the first step of glutamate degradation towards pyruvate, and it was therefore added to further stimulate microbial butyrate formation. Capsules were coated with a methyl methacrylate-based polymer (EUDRAGUARD^®^ biotic; E1207, Evonik, Darmstadt, Germany) with pH-dependent solubility to enable a colon-specific delivery of the capsules. Resistance of the coated capsules towards gastric and small intestinal conditions was confirmed by means of the standardized gastrointestinal in vitro model SHIME^®^ (ProDigest and Ghent University, Ghent, Belgium). Two capsules were taken daily with water (one in the morning and one in the evening) for 4 weeks. Compliance of product intake was calculated from returned capsules.

#### 2.2.4. Sampling and Assessments

Stool samples were collected by subjects at home within maximum 48 h prior to the study visits. Stool samples were directly frozen at subjects’ home after collection (within 10 min) and returned to study site in a frozen state. The stool biomarkers SCFAs (acetate, butyrate, propionate) were analyzed by gas chromatography at Enterosan Labor LS SE & Co. KG, an accredited lab (Bad Bocklet-Großenbrach, Germany). Blood sampling was performed at each visit after at least 10 h overnight fast. For the assessment of total GLP-1 and PYY, DPP-IV and AEBSF inhibitor was added to the EDTA-plasma tube prior to blood collection. Total GLP-1 and PYY were analyzed in plasma utilizing ELISA technique with technical duplicates for each sample according to the manufacturers protocol (Merck Millipore EZGLP1T-36K for total GLP-1 and EZHPYYT66K for PYY). Lipid status (triglycerides, total cholesterol, HDL- and LDL-cholesterol) and further safety blood routine measures were determined in an accredited routine lab at Synlab Medizinisches Versorgungszentrum Leinfelden (Leinfelden, Germany). Stool frequency and consistency by means of the Bristol stool scale were reported daily in a diary. Fiber intake was estimated with a 3-days food protocol and evaluated with EBISpro (Dr. Ehrhardt, Willstätt-Legelshurst, Germany) based on the German Nutrient Data Base.

##### DNA Extraction and Sequencing from Stool Samples

DNA was isolated from approximately 200 mg stool sample using the QIAamp Fast DNA Stool Mini Kit (51604) with a prior bead beating step of the stool samples in InhibitEX^®^ Buffer (Qiagen, Venlo, Netherlands). To characterize the microbial community of the stool samples, 16S rRNA gene amplicon sequencing based on the Illumina protocol for the construction of 16S metagenomic sequencing libraries (Illumina Inc., 2014, San Diego, CA, USA) was performed by Microsynth AG (Balgach, Switzerland). A two-step PCR with the bacterial primers 341F_ill and 802R_ill was used to amplify the third and fourth hypervariable regions of the 16S rRNA gene. Next, amplicon libraries were constructed with the Nextera DNA Library Prep kit. The DNA was purified, pooled equimolarly and the Amplicon libraries were sequenced on an Illumina MiSeq platform (Illumina, San Diego, CA, USA) using the Illumina paired-end protocol (250 bp paired end read, V2 chemistry). After sequencing, libraries for each sequencing lane and sample were demultiplexed and the raw reads were sorted by the inline barcodes. Next, adapters and primer sequences were trimmed.

##### Bioinformatic Processing and Data Cleaning

The obtained Illumina-sequenced paired-end.fasta files (demultiplexed, trimmed and clipped as described above) were further processed with the package ´dada2´ v1.18.0 [21] with default settings as implemented in R version 4.0.3 [22]. Taxonomy was called using the SILVA reference database [23]. The output of the ´dada2´ pipeline is an amplicon sequence variant (ASV) table, which is a higher-resolution analogue of the traditional OTU table, and which records the exact abundance of each amplicon sequence variants occurring in each sample [21]. 

After bioinformatic processing, we performed consecutive data cleaning and filtering steps on the ASV table using R version 3.6.3 [22]. We removed all ASVs with less than 10 read counts and retained only those ASVs in the data set that occurred in at least two samples. We also discarded all ASVs that could not be classified at phylum level. Data cleaning and filtering reduced the number of ASVs from initial 2503 to 1619 ASVs with an average read count of 43,443 (min: 22,407, max: 60,041). Rarefaction with the function *rrarefy()* from the R package ´vegan´ v2.5.7 (https://cran.r-project.org/web/packages/vegan/index.html, accessed on 31 August 2021) to 22,382 read counts per sample reduced the number of ASVs to 1611.

#### 2.2.5. Safety

Adverse events (AE) and concomitant medication were documented throughout the study period. Furthermore, safety blood routine parameters and vital signs were determined. Tolerability was assessed at visit 3 at the end of supplementation period. Subjects rated overall tolerability as “well tolerated”, “slightly unpleasant” or “very unpleasant”. Additional comments were recorded separately.

#### 2.2.6. Statistical Analysis

##### Clinical Parameters

Data of the ‘proof of concept’ study were evaluated in an exploratory manner. Pairwise testing between baseline and 2 weeks of intervention as well as baseline and 4 weeks of intervention was performed. Non-normality was evaluated with Shapiro-Wilk test (*p* < 0.05). All statistical tests were performed two-sided, with a significance level of 0.05. Statistical analysis and graphs were generated using GraphPad Prism (Version 5.04). Within figures, mean levels with 95% confidence interval are depicted. In addition, clinical parameters were analyzed using Spearman’s rank correlation and, in case of ties, with Kendall’s rank correlation.

##### Biostatistical Analyses of the Microbial Community Data

We assessed alpha diversity based on ASVs per sample via the two indices Richness and Pielou’s index of Evenness as implemented in the package ‘vegan’.

To analyze beta diversity, we performed various multivariate statistics implemented in the package ‘vegan’ such as non-metric multidimensional scaling (NMDS) using the function *metaMDS()* with default settings and hierarchical cluster analysis (average linkage with a Bray-Curtis distance). To analyze the impact of subject and treatment on the overall microbial community composition, we performed permutational multivariate analysis of variance (perMANOVA) either with subject or treatment with subject as block factor (strata) using the *adonis()* function and a Bray-Curtis distance from the package ‘vegan’. To explain the distribution of samples in the NMDS, the relationship between alpha diversity indices and the NMDS axes MDS1 and MDS2 were analyzed with Spearman’s rank correlation and, in case of ties, with Kendall’s rank correlation.

Taxonomic profiling was performed as described in [24]. Shortly, the read counts were summed up (either per subject or per timepoint). The read counts of all ASVs belonging to one specific taxon were summed up and normalized by the total number of read counts per sample yielding relative proportions for each taxon. Each taxonomic level was then deduced from the normalized read counts by summing up the classifications on different levels.

For exploratory data analysis, we performed various types of analyses. To determine those taxa on species level, of which the abundance changed due to the intervention with the probiotic, we used Friedman rank sum tests with ‘subject’ as block. We corrected the obtained *p*-values for the false discovery rate as established by Benjamini et al. [25] to account for false positives. To determine which taxa (based on ASVs) correlated to butyrate levels in the stool, we performed multiple regression analyses and corrected *p*-values for the false discovery rate as described above.

For all biostatistical analyses, we used R version 3.6.3 [22]. The boxplots shown in this paper consist of the median (bold horizontal line), the boxes (interquartile range), and the whiskers, which extend to max. 1.5 times the interquartile range. Outliers are depicted as circles.

## 3. Results

### 3.1. Effects of Amino Acids, Peptides and Bacillus Strains in an In Vitro Colonic Screening Platform

To enhance microbial butyrate production capacity in vitro and in vivo, we conceived applying a probiotic microbiome modulator which leads to the expansion of known butyrate-producing taxa executing the acetyl-CoA butyrate pathway [11], as exemplified before for spore-forming *Bacillus* strains [22,23,24]. We compared four probiotic strains of the species *Bacillus subtilis, Bacillus licheniformis*, and *Bacillus amyloliquefaciens* in the I-screen model. *Bacillus subtilis* DSM 32315 increased butyrate concentration by +3.0 mM at *t* = 24 h in comparison to control; another *Bacillus subtilis* strain 2, a *Bacillus licheniformis* and a *Bacillus amyloliquefaciens* strain tested in parallel changed butyrate concentration by −3.0 mM, +1.0 mM; and +4.8 mM, respectively. We also determined changes of branched-chain fatty acids (BCFA) as follows for iso-valerate: *Bacillus subtilis* DSM 32315 −0.7 mM; *Bacillus subtilis* strain 2–0.8 mM; *Bacillus licheniformis* +2.7 mM; *Bacillus amyloliquefaciens* +2.3 mM, and for iso-butyrate: *Bacillus subtilis* DSM 32315 −0.5 mM; *Bacillus subtilis* strain 2 −0.5 mM; *Bacillus licheniformis* +0.5 mM; *Bacillus amyloliquefaciens* +0.4 mM. As the profile of SCFA and BCFA modulation was most favourable for *Bacillus subtilis DSM* 32315, this strain was used for all further experiments. In line with elevated butyrate levels, the proportion of the clostridium XIVa group was 8% in *Bacillus subtilis* DSM 32315-treated samples, compared to 5% in controls.

**Figure 2:**
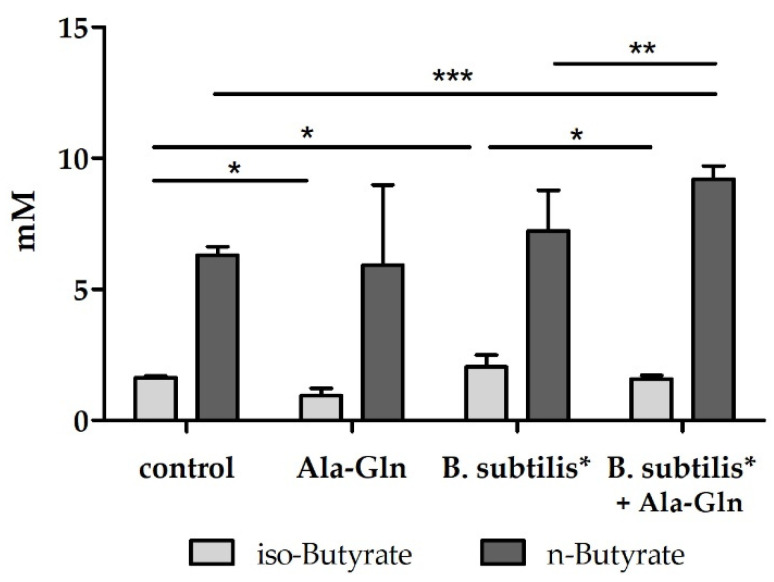
Fecal concentration of n- and iso-butyrate in I-screen experiments supplemented with Ala-Gln ± *Bacillus subtilis* DSM 32315 for 24 h, data shown as means ± 95% CI of triplicate experiments. Significance levels are shown vs. control * *p* < 0.05, ** *p* < 0.01, *** *p* < 0.001 as well as the synergistic effects by combination of *Bacillus subtilis* DSM 32315 with the dipeptide L-Alanyl-L-Glutamine vs. *Bacillus subtilis* DSM 32315.

Next, we probed the synbiotic potential of combinations of several amino acids and dipeptides in conjunction with *Bacillus subtilis* DSM 32315 with butyrate and BCFA levels as readouts. The strongest synergy was found for the dipeptide Ala-Gln, which enhanced *Bacillus subtilis* DSM 32315-driven increase in butyrate (Figure 2) and concomitantly decreased levels of iso-butyrate and iso-valerate, both BCFAs being linked to possibly adverse effects and ageing [26,27]. Moreover, the microbiota was shifted to increased abundance of butyrate producers (*Clostridium* XIVa group), and this effect was more pronounced than for the single treatments (not shown).

### 3.2. Human Study

#### 3.2.1. Subject Characteristics

The investigated study population was a non-smoking study group of busy men with health unconscious eating patterns. Baseline conditions of the study participants are summarized in Table 1. Over the study period, subjects were instructed to keep their normal lifestyle, nutritional and sporting habits. No changes were reported over the study period. With respect to diet, a 3-day food protocol after 2 and 4 weeks of intervention confirmed the comparable fiber and calorie intake throughout the study.

#### 3.2.2. Stool Biomarker: SCFAs

**Figure 3:**
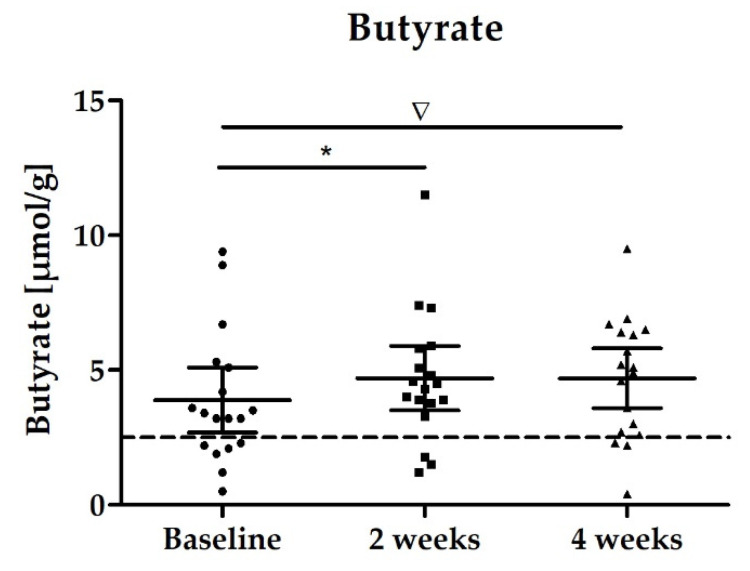
Distribution of fecal butyrate levels [µmol/g]; scatter plot with mean ± 95% CI; * *p* = 0.0278; ∇ *p* = 0.0728.

The main focus of the study was to determine the effects of the study product on the change in levels of SCFAs in feces. While the overall levels of SCFAs did not change, we observed an increase of 21% in butyrate levels over the study period, which was significant after 2 weeks and an increase by trend after 4 weeks (Table 2, Figure 3). It is worth mentioning that six of 18 subjects had butyrate levels below reference range at baseline assessment. At the end of intervention after 4 weeks, only three of 18 subjects had butyrate levels below reference range. On average, there were no significant changes in propionate and acetate levels in stool samples during the study intervention (Table 2).

#### 3.2.3. Microbiome Analysis

**Figure 4:**
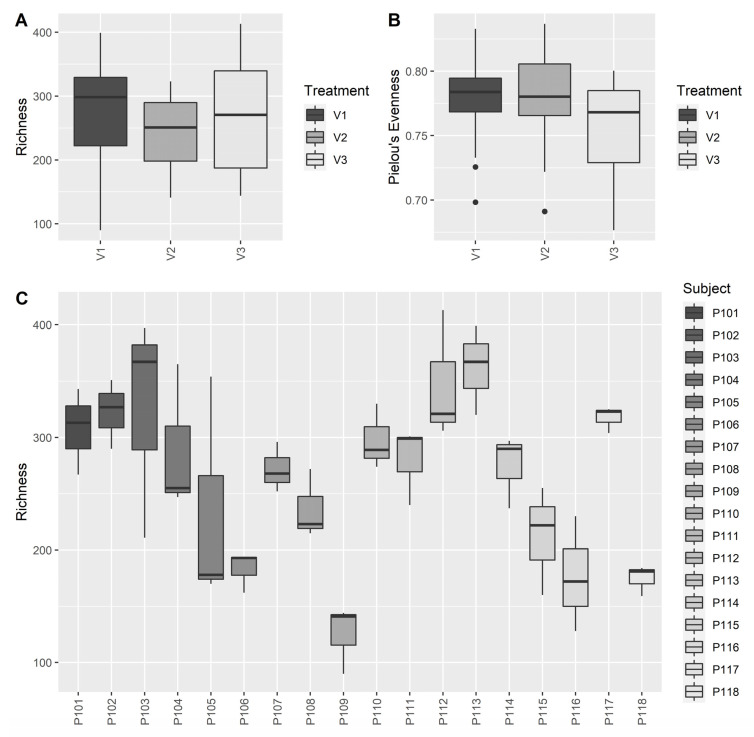
Alpha diversity of stool samples taken at V1 (baseline), V2 (after 2 weeks), and V3 (after 4 weeks) from *n* = 18 subjects (*N* = 54) samples. (**A**) Richness and (**B**) Pielou’s Index of Evenness over time and (**C**) Richness of subjects (based on the absolute abundance of ASVs per sample).

We analyzed the alpha diversity over time. As expected, the observed richness and evenness varied hugely between the subjects and particularly, the richness varied as much as by factor 4.6 between the subjects with the highest and the lowest diversity (Figure 4C, min: 90, max: 413). While the product intervention did not affect richness over time, thus the abundance of ASVs per sample (Friedman rank sum test, Q > 0.05), the Pielou’s Index of Evenness decreased (Friedman rank sum test, Q < 0.05), indicating that the overall proportion of different taxa shifted slightly within samples.

Overall, subjects differed significantly with respect to their microbial community composition (perMANOVA, F_17, 53_ = 10.442, R² = 0.8313, *p* = 0.001). And even though treatment-related effects were not visually apparent (Figure 4A,B), the intervention with the probiotic affected the microbial community composition over time (stratified perMANOVA with subject as block, F_2, 53_ = 0.3062, R² = 0.0119, *p* = 0.0280).

**Figure 5:**
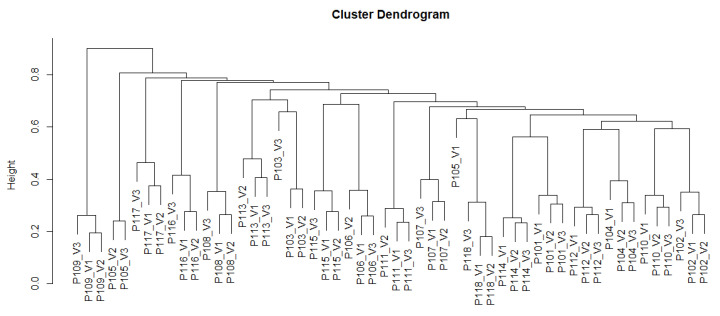
Hierarchical cluster analysis (with Bray-Curtis distance) based on ASVs composition of stool samples taken at V1 (baseline), V2 (after 2 weeks), and V3 (after 4 weeks) from *n* = 18 subjects (*N* = 54 samples).

**Figure 6:**
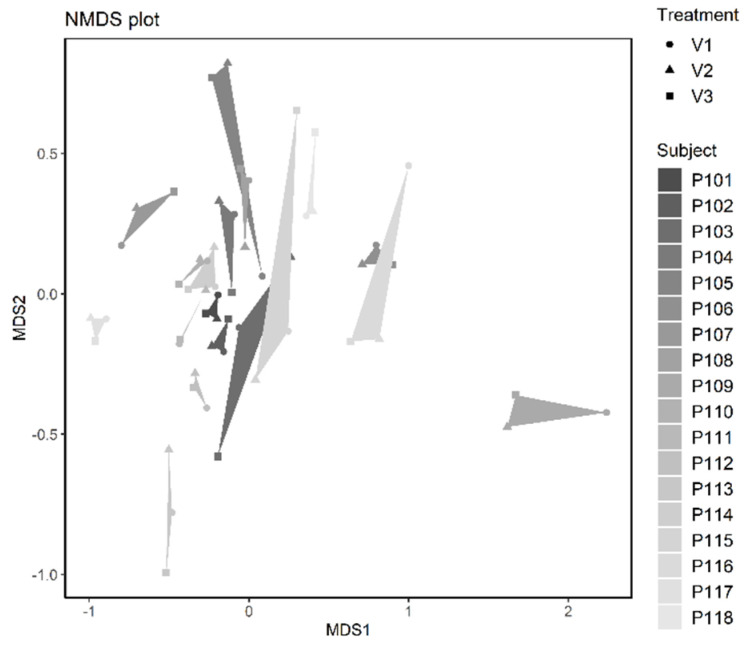
Ordination plot of an nMDS analysis (using Bray-Curtis distance) of the microbial community in stool samples taken at V1 (baseline), V2 (after 2 weeks), and V3 (after 4 weeks) from *n* = 18 subjects (*N* = 54 samples).

Hierarchical clustering and non-metric multidimensional scaling showed that the microbial community composition of each subject was rather consistent over the period of the product intervention (Figure 5 and Figure 6). Nevertheless, the intervention with the product had an individual impact on the microbial community composition of each subject, in many cases leading to the most pronounced changes between baseline and 2 weeks or otherwise between the 2 week’s and 4 week’s sampling. For all subjects, the three samples per subject taken at baseline, after 2 weeks and after 4 weeks were clustered together, indicating that each subject harbored a rather consistent microbial community (Figure 5). In the NMDS (stress = 0.1568), asv richness mainly affected the distribution of subjects both along axis MDS1 (Kendall´s rank correlation, tau = −0.4757, z = −5.0735, *p* < 0.0001) and along axis MDS2 (tau = −0.3652, z = −3.8947, *p* < 0.0001), while treatment (the sampling timepoint coded as numeric factor) did not correlate with any of the axes (MDS1: tau = −0.0678, z = −0.6349, *p* = 0.5255; MDS2: tau = −0.0051, z = −0.0476, *p* = 0.9620).

Taxonomic profiling revealed that of the 195 genera found in this study, only the following eleven genera—*Bacteroides*, *Blautia*, *Alistipes*, *Bifidobacterium*, *Coprococcus*, *Lachnoclostridium*, *Dorea*, *Anaerostipes*, *Ruminococcus torques* group, *Butyricicoccus*, *Odoribacter*, and one unclassified genus—were present in all subjects at all sampling times and thus constituted the core microbiota of this study population. Of the 282 species found in this study, only six species, namely *Bacteroides* unclassified, *Blautia* unclassified, *Lachnoclostridium* unclassified, *Coprococcus comes*, *Ruminococcus torques* group, and *Butyricicoccus* unclassified, as well as two unclassified taxa, occurred in all subjects at all sampling times.

Differential analyses revealed that the abundance of 20 species changed significantly over time (Friedman rank sum test, all *p* < 0.05, Q = n.s., see Table S1), of which *Faecalibacterium prausnitzii* represented by five different ASVs was the third most abundant taxon in this study.

To test whether any of these taxa might be responsible for changes in butyrate levels in the stool, we furthermore performed multiple correlation analyses. Of the five different ASVs classified as *F. prausnitzii*, only one strain correlated significantly with the determined butyrate levels in the stool (Kendall´s rank correlation, tau = 0.3465, z = 3.6674, *p* = 0.0002; treatment effect of this strain: Friedman rank sum test with subject as block, *p* = 0.0619), while the others did not (Kendall´s rank correlation, *p* = n.s., sum of all *F. prausnitzii* strains: tau = 0.1769, z = 1.8808, *p* = 0.0600).

#### 3.2.4. Blood Biomarkers: Total GLP-1, PYY, Fasting Blood Glucose and Lipid Status

**Figure 7:**
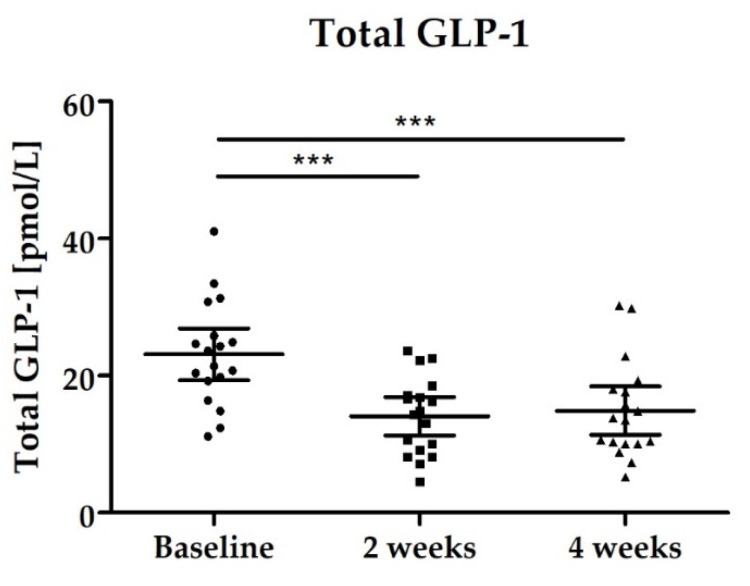
Distribution of Total GLP-1 levels [pmol/L]; scatter plot with mean ± 95% CI; Baseline vs. 2 weeks *** *p* < 0.001 (paired *t*-test); Baseline vs. 4 weeks *** *p* < 0.001 (paired *t*-test).

**Figure 8:**
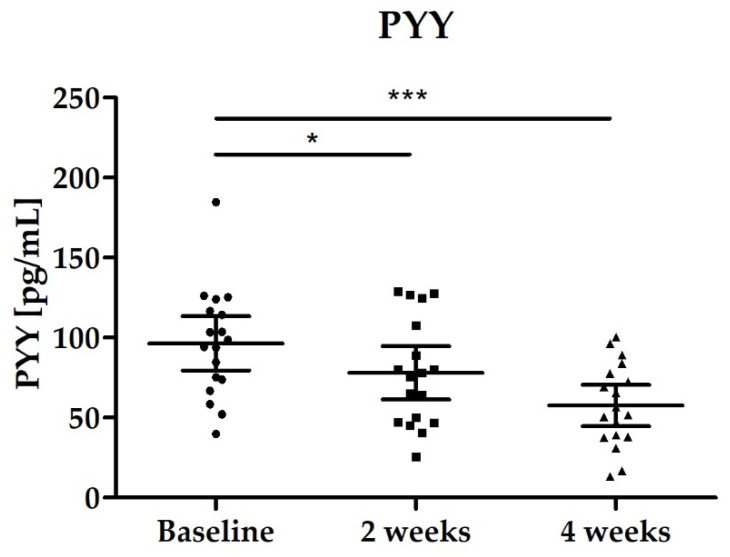
Distribution of PYY levels [pg/mL]; scatter plot with mean ± 95% CI; Baseline vs. 2 weeks * *p* = 0.0139 (paired *t*-test); Baseline vs. 4 weeks *** *p* < 0.001 (paired *t*-test).

Both hormones (total GLP-1, PYY) showed a significant decrease during the study. All samples were collected under fasting conditions. Total GLP-1 levels decreased significantly between baseline and end of intervention after 4 weeks (*p* < 0.0001) which was already confirmed after 2 weeks of intervention (Figure 7). The significant decrease was also confirmed for PYY, see Figure 8 and Table 3.

**Figure 9:**
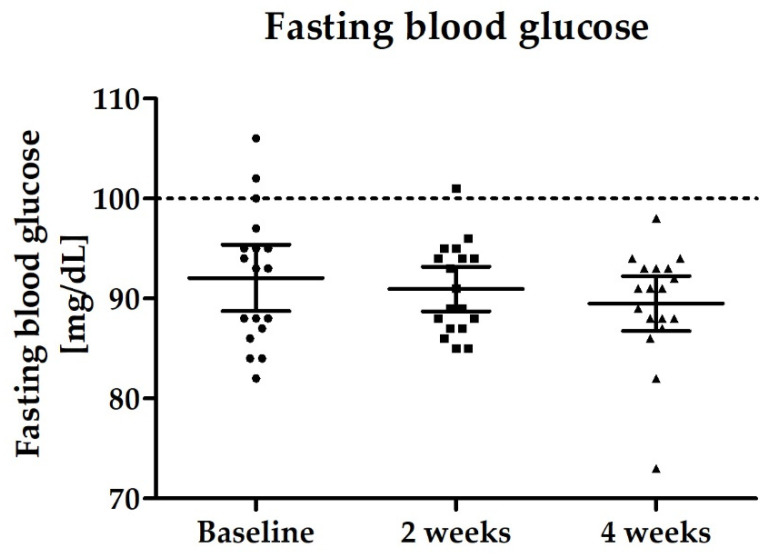
Distribution of fasting blood glucose levels [mg/dL]; scatter plot with mean ± 95% CI; line indicates upper normal reference range <100 mg/dL.

In the light of the significantly altered incretin concentrations during the course of the study, it is worth mentioning, that also for fasting glucose concentration a slight reduction was observed over the study period, but differences were not statistically significant (*p* = 0.2965) (see Figure 9).

**Figure 10:**
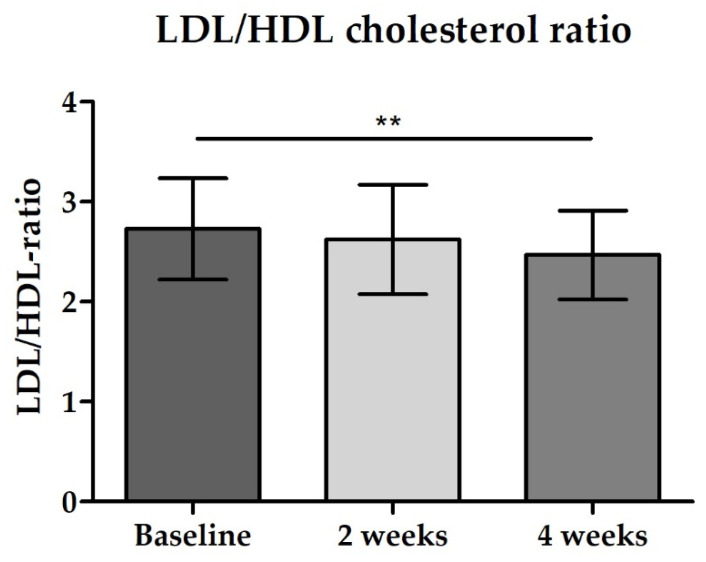
Distribution of LDL/HDL cholesterol ratio; bar chart with mean ± 95% CI; Baseline vs. 4 weeks ** *p* = 0.0022 (paired *t*-test).

The intake of study products also affected lipid status in blood. Total cholesterol levels as well as LDL cholesterol levels decreased significantly between baseline and end of intervention (see Table 4). In contrast, a slight increase was observed for HDL-cholesterol resulting in a significant reduction in LDL/HDL ratio after the 4-week intervention (*p* = 0.0022, Figure 10). Moreover, triglycerides levels did not change significantly over the study period (see Table 4).

Correlation analyses of blood and fecal parameters showed that GLP-1 and PYY correlated positively to each other (Spearman rank correlation, rho = 0.5312, S = 12298, *p* < 0.001). While GLP-1 was inversely correlated with HDL in the blood (Kendall’s rankcorrelation, tau = −0.2381, z = −2.5043, *p* = 0.0123), PYY was inversely correlated to butyrate levels in the stool (Kendall’s rank correlation, tau = −0.1825, z = −1.9405, *p* = 0.0523). Both the total cholesterol and LDL-cholesterol correlated positively to the fasting glucose levels (Kendall’s rank correlation, total cholesterol: tau = 0.2482, z = 2.5646, *p* = 0.0103, LDL-cholesterol: tau = 0.2040, z = 2.1073, *p* = 0.0351).

#### 3.2.5. Safety Assessment and Compliance

The safety of the strain was tested by means of a cytotoxicity test, assessment of antimicrobial susceptibility, and genome analyses according to the EFSA criteria (‘Guidance on the characterization of microorganisms used as feed additives or as production organisms’) for *Bacillus* strains. No safety risk was detected.

During the clinical study, compliance of intake of the study product in the study group was high with a mean of 97.6%. Tolerability was assessed at the end of the intervention phase. The study product was well tolerated by 15 subjects, three subjects rated tolerability as slightly unpleasant and none of the subjects rated tolerability as very unpleasant. The minor limitation of the tolerability assessment was justified by subjects with “capsules were too big and difficult to swallow”, “heartburn or acidic burping in rare cases” (2x over the intervention period of 4 weeks) and one subject reported a “higher stool frequency” (increase from 6.5 stools/weeks during run-in phase to 9.0 stools/week during week 3 + 4 of intervention). With respect to stool frequency and stool consistency (classified by Bristol Stool Scale) in the whole study group, no significant changes were observed (Frequency: Baseline 7.2 stools/week (95%CI: 5.3–9.0), end of intervention 6.9 stools/week (95%CI: 5.4–8.5). Consistency: Baseline 3.7 (95%CI: 3.3–4.2), end of intervention 3.5 (95%CI: 3.1–3.9).

No safety issues were identified checking the blood routine parameters and vital signs.

## 4. Discussion

Here we described the development of a novel synbiotic formulation targeted at modulating human gut microbiota towards butyrate production. Butyrate has emerged as one important mediator of the impact of gut microbiota on human health [11,28,29]. In consequence, efforts have been made to increase its availability or formation in the gut through prebiotic fibers, probiotics, and direct application of butyrate or precursors thereof [28]. Although several butyrate-producing bacteria have been isolated and studied with promising outcomes in preclinical settings [13,28], there is still a long way to go to determine their safety and efficacy in humans.

Few trials with *Akkermansia muciniphila*, *Faecalibacterium prausnitzii*, *Clostridium butyricum*, *Clostridium beijerinckii*, and *Eubacterium hallii* have been conducted, and in none of them fecal butyrate levels were significantly increased. Moreover, these strains are strictly anaerobic, and this poses a challenge not only for industrial production processes, but also for their stability during storage and the passage through the gastrointestinal tract. The genus *Bacillus* on the other hand comprises aerotolerant/microaerophilic spore-forming bacteria, which therefore have the advantage of technical robustness. Commonly applied *Bacillus* probiotics with a long history of safe use in humans belong to the species *Bacillus subtilis*, *Bacillus coagulans, Bacillus licheniformis* [30]. While these *Bacillus* species are not butyrate producers themselves, they appear to have indirect, modulating effects on the gut microbiota, triggering butyrate production in other taxa. This has been exemplified by studies with other *Bacillus* strains. Our results of *Bacillus subtilis* DSM 32315 inducing butyrate levels in, but not without a complex microbiota add to these findings (see Figure 2). The indirect butyrogenic effect is difficult to uncover completely; we may speculate though that lactate, which is produced by *Bacillus subtilis* DSM 32315, serves in cross feeding reactions as substrate for butyrate producers. Interestingly, the microbiota composition of infants has been shown to transition from a high lactate/high formate towards a high propionate/high butyrate profile within 12 to 24 months after birth [31]. This functional change was paralleled by an expansion of *Clostridiales* phylotypes, many of which possess the acetyl-CoA pathway. This phase of lactate/formate elevation appears to be limited to this very early period in life, and we reason that *Bacillus subtilis* DSM 32315-derived lactate (not shown) contributed to the functional and compositional changes of adult microbiota described in our study. In line with that, the butyrogenic effect of *Bacillus subtilis* DSM 32315 was associated with shifts in the evenness of the microbial community, but not with increases in diversity during the human study (Figure 4), indicating that particular populations increased and others decreased in abundance due to the intervention. While each subject harbored a very individual microbial community (Figure 6), our product affected the relative proportion of twenty taxa over time, and particularly increased levels of *Faecalibacterium prausnitzii*, a well-known producer of butyrate [32]. Furthermore, the abundance of one out of the five determined potential *Faecalibacterium prausnitzii* strains positively correlated to butyrate levels in stool. As most of the other taxa affected by the synbiotic either did not show a significant correlation to butyrate levels in the stool or otherwise were of much less abundance than *F. prausnitzii* (Table S1), our results suggest that *F. prausnitzii* might be the main taxon responsible for the observed increased butyrate levels over time (Figure 3), which would also be in accordance with the results of previous studies [15,16].

Even though we found that both butyrate levels and the abundance of *Faecalibacterium prausnitzii* were significantly affected by the treatment intervention, in the face of a small sample size investigated in this pilot study and taking into account the huge inter-individual variability of the microbiota as well as the metabolites, the significance level was missed after 4 weeks but still a difference per trend was seen for butyrate. We assume fecal butyrate concentrations might have come to a (subject-specific) plateau after 2 weeks of supplementation, wherein the microbiota composition has formed a new equilibrium (still with huge inter-individual differences).

Synbiotic approaches usually combine probiotics with fibers to achieve synergistic effects on SCFA production and/or expansion of *Lactobacilli* and *Bifidobacteria*. We here broadened the term “synbiotic” towards inclusion of amino acids and peptides as substrates for microbial metabolization. The application of fiber and FODMAPs is limited or not desirable under certain medical conditions or dietary restrictions. Also, amino acids can offer a targeted approach towards specific metabolization routes, e.g., the acetyl-CoA butyrate pathway. The butyrogenic activity of Ala-Gln can be attributed to glutamine, which hydrolyzes to glutamic acid and is then catabolized to butyric acid, while the alanine residue not only increases stability of the dipeptide, but may also serve as a spore germination trigger [33] and thereby supports *Bacillus subtilis* function after its release from the capsule into the colonic lumen. Both Ala-Gln and *Bacillus subtilis* DSM 32315 individually showed a somewhat similar modulation of the gut microbiota in our intestinal screening assays, i.e., expansion of the clostridium group XIV. The extent of components that we screened for effects on gut microbiota activity (SCFA production) and composition was limited, especially for the number of strains tested. It therefore remains to be determined if related strains, at least from the same species *Bacillus subtilis*, have similar microbiome-modulating effects, and in that sense the composition of the resident microbiota would be an important determinant of the effect of Ala-Gln or related peptides if administered/released alone. Regardless, it can be speculated that a synbiotic approach, as exemplified here, may reduce the inter-individual responsiveness towards a single component intervention and may result in more predictable outcomes.

Our human study was conducted as proof-of-concept study for elevation of fecal butyrate as primary outcome and as an exploratory study with multiple clinical parameters. The strongest effects were seen for the satiety hormones GLP-1 and PYY as well as plasma lipids and hints on modulation of fasting glucose levels. To our knowledge, an association of *Bacillus subtilis* DSM 32315 or Ala-Gln with these blood markers has not been described before. As HDL-cholesterol and HDL/LDL ratio were rather positively affected by the intervention, the inverse correlation to GLP-1 might indicate that the observed decrease could also be interpreted in a positive manner. However, the causal relationship between HDL-cholesterol and GLP-1 levels in the blood needs to be further investigated. The inverse relation between butyrate levels and PYY might be similarly interpreted as a positive outcome of the intervention. The positive correlation of total cholesterol and LDL-cholesterol to fasting glucose is interesting indicating the overall metabolic impact in some subjects. Thus, further studies should include information on BMI and changes in body weight to potentially shed a light into physiological implications of blood lipid status and fasting glucose level in particular in study subjects in the prediabetic stage.

Connections between gut microbiota composition as well as probiotic supplements and traits of the metabolic syndrome have been described before [34]. Recently, Wu et al. found out that several butyrate producers and functional potential for butyrate production were decreased both in prediabetes and in type 2 diabetes (T2D) [35], confirming earlier findings by Zhang et al. on reduced butyrate-production capacity during the progression of glucose intolerance [36]. T2D in particular appears to (adversely) affect gut microbiome composition and functionality [29]; a meta-analysis of RCT revealed that fiber intake in diabetics had no significant effect on butyrate levels [37], which is in contrast to effects of fibers in healthy individuals [38]. In line with that is another trial using a multi-strain probiotic supplement which induced microbial butyrate-producing pathways in diabetics and prediabetics, but only in those participants who were also treated with metformin [39]. Despite the normoglycemic study collective, but with three subjects in the prediabetic range during baseline, it is worth mentioning that over the study period a modulation of fasting glucose levels was seen whereas all participants were in the normoglycemic range post intervention. It might therefore be worthwhile to test the efficacy of our evidence-based synbiotic approach in larger cohorts with impaired glucose tolerance as well as type 2 diabetes and to determine whether it can act as an adjuvant therapy for metformin.

The significant reductions of GLP-1 and PYY observed in our study were counterintuitive, given the reductions in fasting glucose and cholesterol levels. Importantly, most studies on satiety hormones assess their post-prandial levels or release, which is attenuated in metabolic disorders compared to a healthy status. In the fasting state, however, people with normal glucose tolerance had significantly lower GLP-1 plasma concentrations than people with T2D [40,41]. In a case-control study, GLP-1 levels were positively correlated with metabolic syndrome traits [41], whereas fasting GLP-1 was reduced in obese subjects following weight-loss diets [42,43]. However, weight was not a parameter of our study and participants were instructed not to change their lifestyle including dietary habits during the course of the study.

Probiotics have been applied to mitigate cardiovascular disease risk factors, with circulating cholesterol levels as a particular target. The clinical evidence for cholesterol reduction by probiotic supplementation is limited so far, with some improvements seen in diabetic and hypercholesterolemic patients [44,45], but not in subjects with normal or slightly elevated cholesterol levels [46]. Notably, we here observed relatively small but significant reductions of LDL- and total cholesterol levels after the 4-week period in normocholesterolemic subjects. The mode of action of our synbiotic approach on lipid metabolism needs to be elucidated further. Anti-cholesterolemic activity of probiotics can be caused by e.g., bile salt hydrolysis (BSH), interference with hepatic de novo synthesis of lipids via modulation of SCFA or satiety hormones [47]. The BSH activity of some bacterial strains lowers the enterohepatic cycling of bile salt conjugates, compensated by *de novo* synthesis from cholesterol in the liver, resulting in reduced plasma cholesterol and lipoprotein levels. Genome analysis of *Bacillus subtilis* DSM 32315 does not indicate that the strain carries BSH activity, however, gut residents with BSH activity may have expanded during synbiotic supplementation. Overall, this pilot study characterizes our synbiotic composition as a novel tool for inducing fecal butyrate levels, with indicative findings on modulation of glucose and lipid metabolism. One limitation of our study is that the study product contained also additional ingredients (vitamins, zinc, curcuma, and green tea extracts), and therefore the effects observed here cannot solely be assigned to the synbiotic combination. Further comparative assessments of the synbiotic formulation with and without additional ingredients are warranted to confirm our findings and to reveal the mode(s) of action(s) of our synbiotic approach.

## 5. Conclusions

We identified a new synbiotic combination based on a robust probiotic strain *Bacillus subtilis* DSM 32315 and the dipeptide L-alanyl-L-glutamine that can modulate the human colonic microbiota towards a pro-butyrogenic shift in its composition and activity, and to combine it with a stable, non-fiber substrate that is selectively used for butyrate production.

In conclusion, our study indicates that this synbiotic composition may provide an effective and safe tool for stimulation of intestinal butyrate production with effects on e.g., lipid and glucose homeostasis. Further investigations in larger cohorts are warranted to confirm and expand these findings.

## 6. Patents

PCT/EP2019/060158.

## Figures and Tables

**Table 1:** Demographic data and baseline characteristics (at screening).

Variable	Study Population (*n* = 18)
	Mean (95% CI)
Age [years]	31.7 (29.5–33.9)
BMI [kg/m²]	24.8 (23.5–26.1)
Cholesterol [mg/dL]	180.2 (166.9–193.4)
LDL-cholesterol [mg/dL]	123.9 (106.1–141.7)
GPT [U/L]	27.7 (21.8–33.6)
GOT [U/L]	19.3 (16.9–21.8)
Fasting blood glucose [mg/dL]	92.9 (90.4–95.4)
Calorie intake	3005 (1738–4272)
Fiber intake [g/d]	18.5 (14.6–22.38)
Stool frequency [stools/week]	7.2 (5.3–9.0)
Stool consistency [Bristol]	3.7 (3.3–4.2)
Systolic blood pressure	130 (125–136)
Diastolic blood pressure	77 (74–81)

BMI: body mass index; BP: blood pressure; LDL: low-density lipoprotein, GPT: glutamate pyruvic transaminase, GOT: glutamic oxaloacetic transaminase.

**Table 2:** SCFAs in fecal samples at baseline, after 2 weeks and after 4 weeks of intervention (*n* = 18).

Variables	Baseline	2 Weeks	4 Weeks
	Mean (95% CI)	Mean (95% CI)	Mean (95% CI)
Total SCFA [µmol/g]	34.72 (28.55–40.88)	37.55 (31.22–43.88)	35.06 (29.23–40.88)
Butyrate [µmol/g]	3.88 (2.68–5.09)	4.70 (3.50–5.90) *p* = 0.0278 *	4.70 (3.59–5.81) *p* = 0.0728 *
Propionate [µmol/g]	6.16 (4.51–7.81)	6.45 (4.55–8.35)	6.24 (4.71–7.78)
Acetate [µmol/g]	24.64 (20.70–28.58)	26.36 (22.21–30.51)	24.12 (20.09–28.15)

* Wilcoxon signed rank test in comparison to baseline assessment.

**Table 3:** Gut hormones for appetite control (total GLP-1, PYY) at baseline, after 2 weeks and after 4 weeks of intervention (*n* = 18).

Variables	Baseline	2 Weeks	4 Weeks
	Mean (95% CI)	Mean (95% CI)	Mean (95% CI)
Total GLP-1 [pmol/L]	23.11 (19.33–26.88)	14.09 (11.29–16.89) *p* < 0.0001	14.89 (11.37–18.42) *p* < 0.0001
PYY [pmol/L]	96.44 (79.53–113.40)	78.04 (61.43–94.65) *p* = 0.0139	57.52 (44.60–70.44) *p* < 0.0001

**Table 4:** Lipid status and fasting blood glucose levels at baseline, after 2 weeks and after 4 weeks of intervention (*n* = 18).

Variables	Baseline	2 Weeks	4 Weeks
	Mean (95% CI)	Mean (95% CI)	Mean (95% CI)
Total cholesterol [mg/dL]	179.3 (164.5–194.2)	172.4 (157.5–187.4)	169.10 (155.5–182.7) *p* = 0.0037 *
LDL cholesterol [mg/dL]	120.6 (103.4–137.7)	117.80 (100.3–135.3)	113.10 (95.0–131.2) *p* = 0.0313 *
HDL cholesterol [mg/dL]	46.1 (41.9–50.2)	46.9 (42.7–51.2)	47.3 (42.6–52.1)
Triglycerides [mg/dL]	116.3 (95.0–137.5)	119.4 (98.7–140.2)	124.2 (89.1–159.4)
Fasting blood glucose [mg/dL)	92.1 (88.7–95.4)	90.9 (88.7–93.2)	89.5 (86.8–92.2)

* paired *t*-test.

## Data Availability

The data presented in this study are available on request from the corresponding author. The data are not publicly available due to patenting processes.

## Supplementary Materials

The following are available online at https://www.mdpi.com/article/10.3390/nu14010143/s1. Table S1: Treatment affected the relative proportion of twenty species over time (Friedman rank sum test with subject as block factor, *N* = 54 with *n* = 18 subjects at *n* = 3 timepoints [18]). Taxa were ordered by decreasing median at baseline.
