# Supplementary material for: A Synbiotic Formulation Comprising Bacillus subtilis DSM 32315 and L-Alanyl-L-Glutamine Improves Intestinal Butyrate Levels and Lipid Metabolism in Healthy Humans
Source: Nutrients. 2021 Dec 29;14(1):143. doi: 10.3390/nu14010143 (PMC8747176; doi:10.3390/nu14010143)
Supplement: Supplementary file 1 [file nutrients-14-00143-s001.zip › nutrients-1506053-supplementary.pdf]

A synbiotic formulation comprising *Bacillus subtilis* DSM 32315 and L-alanyl-L-glutamine improves intestinal butyrate levels and lipid metabolism in healthy humans

Table S1. Treatment affected the relative proportion of twenty species over time (Friedman rank sum test with subject as block factor, N = 54 with n = 18 subjects at n = 3 timepoints {baseline, two weeks and four weeks}). Taxa were ordered by decreasing median at baseline.

Taxa	Baseline			2 weeks			4 weeks				
	Median	Min	Max	Median	Min	Max	Median	Min	Max	P-value	FDR corr P-value
Faecalibacterium prausnitzii	1118.5	0	1622	1476	59	2441	1264.5	17	2588	0.0421	0.6492
Ruminococcus unclassified	425.5	0	1975	228.5	0	1165	157	0	953	0.0469	0.6492
Roseburia unclassified	188	0	480	188	3	370	97.5	3	341	0.0308	0.6492
LachnospiraceaeND3007group unclassified	91.5	0	383	141.5	11	226	80.5	0	157	0.0021	0.2958
LachnospiraceaeNK4A136 group bacterium	87.5	0	453	71	0	905	36	0	222	0.0521	0.6492
Lachnoclostridium edouardi	82	0	185	105	7	398	71	0	375	0.0214	0.6492
Monoglobus unclassified	66.5	0	325	69.5	1	355	69.5	1	263	0.0397	0.6492
Dorea unclassified	35	0	175	15	0	152	13	0	119	0.0634	0.6492
Roseburia intestinalis	30	0	1177	59.5	0	761	0	0	1503	0.0183	0.6492
IncertaeSedis unclassified	25.5	2	139	45	10	143	27.5	8	186	0.0124	0.6492
Ruminococcus bromii	17.5	0	898	4.5	0	1199	26	0	2795	0.0187	0.6492
Alistipes obesi	10.5	0	79	12	0	56	15	0	88	0.0498	0.6492
Klebsiella unclassified	3	0	503	0	0	1064	0	0	299	0.0373	0.6492
Bifidobacterium bifidum	1.5	0	141	0	0	179	1	0	214	0.0277	0.6492
Anaerofilum unclassified	0	0	12	0	0	10	0	0	10	0.0013	0.2958
Bacillus unclassified	0	0	1	0	0	3	0	0	53	0.0168	0.6492
Gordonibacter pamelaeae	0	0	0	0	0	3	0	0	8	0.0231	0.6492
Slackia isoflavoniconvertens	0	0	46	0	0	47	5	0	157	0.0354	0.6492
Bacteroides stercoris	0	0	62	0	0	173	0.5	0	91	0.0388	0.6492
Negativibacillus massiliensis	0	0	27	0	0	44	0	0	0	0.0421	0.6492
Bacteroides coprocola	0	0	1206	0	0	2490	0	0	2669	0.0441	0.6492
Colidextribacter massiliensis	0	0	10	0	0	15	3	0	18	0.0458	0.6492
